# Autophagy deactivation is associated with severe prostatic inflammation in patients with lower urinary tract symptoms and benign prostatic hyperplasia

**DOI:** 10.18632/oncotarget.15144

**Published:** 2017-02-07

**Authors:** Cosimo De Nunzio, Simona Giglio, Antonella Stoppacciaro, Mauro Gacci, Roberto Cirombella, Emidio Luciani, Andrea Tubaro, Andrea Vecchione

**Affiliations:** ^1^ Urology Units, Department of Clinical and Molecular Medicine, Ospedale Sant’Andrea, Sapienza University, Rome, Italy; ^2^ Surgical Pathology Units, Department of Clinical and Molecular Medicine, Ospedale Sant’Andrea, Sapienza University, Rome, Italy; ^3^ Department of Cancer Biology and Genetics/CCC, The Ohio State University, Columbus, OH, USA; ^4^ Department of Urology, Careggi Hospital, Firenze, Italy

**Keywords:** autophagy, BPH, LUTS, inflammation, prostate

## Abstract

Autophagy is a conserved evolutionary process that allows cells to maintain macromolecular synthesis and energy homeostasis during starvation and stressful conditions. We prospectively evaluated the relationship between autophagy and prostatic inflammation in a series of transurethral prostatic resection samples. Inflammatory infiltrates were defined according to the standardized classification of chronic prostatitis of the National Institute of Health. The inflammatory score (IS score) was calculated. High IS score was defined as ≥7. Each sample was stained for anti-LC3B and for anti-P62/SQSTM1 and scored. High p62 or LC3B percentage was defined as >25%, whereas low was defined as <25% of cells with dots.

We analyzed 94 specimens. Overall, 18/94 (19%) showed no sign of prostatic inflammation, whereas 76/94 (81%) presented inflammatory infiltrates. Inflammation was mild in 61/76 (80%), moderate/severe in 15/76 (20%). Patients with high p62 percentage were 62/94 (66%) while 32 (34%) showed low p62 percentage. Patients with high LC3B percentage were 37/94 (39%) while 57(61%) showed low LC3B percentage. Overall 42/94 (44%) patients presented a high p62 percentage and concomitant a low LC3B percentage. IS score was significantly higher in patients with a with high p62 percentage (median IS 7 (6/8) vs 5 (3/7); p= 0.04) and in patients with a low LC3B percentage (median IS 7 (6/8) vs 5 (3/7); p= 0.004) when compared to patients with a low p62 percentage or a high LC3B percentage respectively. On multivariate analysis, p62 (OR: 10.1, 95%CI: 2.6-38.6; p= 0,001) and LC3B expression (OR: 0.319; 95%CI: 0.112-0.907; p= 0.032) were independent predictors of a high IS.

Here we present the first evidence of autophagy deregulation in prostatic inflammation. These results raise many questions about the mechanisms mediating the autophagy dysfunction and the links to prostatic inflammation that need to be addressed.

## INTRODUCTION

Lower urinary tract symptoms (LUTS) and Benign Prostatic Hyperplasia (BPH) are highly prevalent in adult males and BPH is the most frequent benign neoplasm in aging men [1]. Although several mechanisms seem to be involved in its development and progression the pathogenesis of this condition is still largely unknown. In the past few years recent evidence clearly suggested the possible role of prostate inflammation in the pathogenesis of LUTS and BPH [2–5].

Autophagy is a highly conserved evolutionary process that is involved in a number of cellular homeostatic processes that allows the cell to maintain macromolecular synthesis and energy homeostasis during starvation and other stressful conditions [6, 7]. Therefore induction of autophagy exerts anti-aging and oncosuppressive functions. A hallmark of autophagy is the formation of the autophagosome (double-membrane cytosolic vacuoles), in which proteins and organelles are imbibed, and then after fusion with lysosomes are degraded. Autophagy is regulated by a series of related genes. In particular two ubiquitin like conjugation system (ATG12-ATG5-ATG16 and ATG8) are crucial for autophagosome formation and cargo recruitment. One of the human homologue of ATG8 (LC3B) represents one of the most widely used markers to monitor this process [8]. Autophagy can be a highly selective process, which is achieved through receptors that are able to interact with the autophagy machinery and to recognize ligand bound cargo. One the best studied receptor is p62, also known as sequestosome (SQSTM1). Since p62 is localized to the autophagosome via LC3-interaction and is constantly degraded by the autophagy–lysosome system, therefore autophagy inhibition leads to the accumulation of p62 positive aggregates [9]. Recent studies [7, 10–12] have shown that in adipocytes or in pancreatic cells the levels of inflammatory gene and cells, and the activation of several inflammatory pathways are influenced by autophagy activation/deactivation. Furthermore there is an intense investigation on targeting autophagy mechanism in several malignancies including prostate cancer. With this knowledge in mind we hypothesized that autophagy could play a role in the prostate cells immune response with a subsequent effect on prostatic inflammation. To this aim we evaluated the relationship between autophagy and prostatic inflammation assessing the expression of autophagy markers P62 and LC3B.

## RESULTS

We analyzed 94 surgical specimens of TransUrethral Resection of Prostate (TURP). Patients’ characteristics are summarized in Table 1. Overall, 18/94 (19%) showed no sign of prostate inflammation at the histology report, whereas 76/94 subjects (81%) presented an inflammatory infiltrate. Inflammation was mild in 61/76 (80%) and moderate/severe in 15/76 (20%). The median inflammatory score was 6 (IQR: 4-7). Overall 60 patients (64%) presented a low inflammatory score (IS) (IS < 7) and 34 (36%) a high inflammatory score (IS ≥ 7).

**Table 1:** Patient's characteristics according to the presence of prostatic inflammation

	Overall	Inflammatory score < 7	Inflammatory score ≥ 7	p
Patients	94	60/94 (64%)	34/94 (36%)	
Age (years)	69,6 ± 6.8 (69; 65-75)	69.6±7.1 (69; 66-75)	69.6±6.5 (71; 65-74.5)	0.779
BMI (kg/m^2^)	22,9±2.8 (22.4; 21-24)	22.7±2.9 (22.6; 20-24)	23.1±2.8 (22.4; 21-24)	0.484
PSA (ng/ml)	6±4 (5.2; 2.7-9.1)	6.5±5.2 (5; 2-9.2)	6±4 (4.7; 3.3-9)	0.865
TRUS volume (ml)	71 ±17 (66; 50-96)	70±28.1 (65; 50-89)	65.8±22.5 (59.5; 50-82)	0.247
IPSS	18.9 ±6.7 (18; 13-24)	17.4±6.1 (16; 13-23)	21.4±7 (24; 16-2721)	0.004
IPSS voiding	9.3±3.7 (10; 6-12)	8.9±3.5 (8; 6-12)	10.1±3.9 (11; 6-12)	0.123
IPSS storage	9 ± 4 (9.5; 6-12)	8.6±4.1 (7.5; (6-12)	10.5 ± 3.9 (11; 9-13)	0.018
Qmax (ml/s)	8.7±2.8 (8.2; 6.1-10.6)	6±1 (6; 4-6)	8.7±2.9 (9; 6-10)	0.756
PVR (ml)	44.6±38.4 (37.5; 17.5-69.2)	33±15 (30; 20-50)	43.7±49.4 (30; 0-88)	0.421

Data are presented as mean ± DS (median; IQR); BMI: body mass index; PSA: prostate specific antigen; TRUS: trans rectal ultrasound; IPSS: International Prostate Symptoms Score; Qmax: maximum urine flow; PVR: post voiding residual.

Patients with prostate inflammation (IS ≥ 7) presented an higher pre-operative International Prostatic Symptom Score (IPSS), when compared to those without an IS < 7 (Table 1).

Patients with high percentage of p62 were 62/94 (66%) while patients with low percentage were 32/94 (34%). Patients with high percentage of LC3B were 37/94 (39%) while patients with low percentage of were 57/94 (61%). Overall 42/94 (44%) patients presented a low percentage of LC3B and a concomitant high percentage of p62 (high p62/low LC3B). Patients with an IS ≥ 7 presented a higher percentage of p62 and a lower percentage of LC3B when compared to patients with a lower IS score (Table 2).

**Table 2:** Patient's characteristics according to the autophagy protein expression and inflammatory score

	Overall	Inflammatory score <7	Inflammatory score ≥7	p
High% p62	62/94 (66%)	29/60 (48%)	31/34 (91%)	0.001
Low% LCB3	57/94 (60%)	31/60 (51%)	26/34 (76%)	0.015
High p62/low BC	42/94 (45%)	18/60 (30%)	24/34 (70%)	0.001

IS score was significantly higher in patients with high percentage of p62 [median IS: 7 (6/8) vs 5 (3/7); p= 0.04] and in patients with a low percentage of LC3B [median IS: 7 (6/8) vs 5 (3/7); p= 0.004] when compared to patients with a low percentage of p62 and a high percentage of LC3B respectively. On multivariate analysis, p62 (OR: 10.1, 95%CI: 2.6-38.6; p= 0,001) and LC3B expression (OR: 0.319; 95%CI: 0.112-0.907; p= 0.03) were independent predictors of a high IS. Age was not associated to an increased risk of inflammatory infiltrates (OR: 1.02; 95%CI: 0.956-1.103; p= 0.46).

## DISCUSSION

The presence of chronic histological inflammation is a well-known finding in biopsy and surgical specimens of prostate tissue in patients with and without lower urinary tract symptoms or prostatitis [3]. Histological inflammation was found in more than 78% of men enrolled in the Reduction by Dutasteride of Prostate Cancer Events trial (REDUCE), demonstrating its ubiquitous nature in aging men, although its relation to histological and clinical BPH is unclear [13]. In our study, most of our patients (81%) with LUTS/BPH treated with a TURP, presented an inflammatory infiltrate. Inflammation was moderate/severe in about 20% of the study population. As recently proposed in several studies [5, 14] investigating the relationship between inflammation and LUTS, prostatic inflammatory infiltrates were defined according to the standardized classification system of chronic prostatitis (CP-CPPS) of the National Institutes of Health (NIH), including the inflammatory score [15]. We confirmed as in previous experiences [16] that a higher IS was observed in about 40% of the study population and it was associated with a more severe IPSS. Patients with prostate inflammation (IS ≥ 7) presented an higher pre-operative IPSS, and particularly an higher IPSS storage subscore. Our data also confirmed a previous experience where metabolic syndrome associated with inflammatory infiltrates through different mostly unknown mechanism significantly increases the risk of an IPSS storage subscore ≥ 4 (OR: 1.782; 95%CI 1.045-3.042; p = 0.030) [17, 18].

Autophagy is a key process for the regular maintenance and disposal of intracellular organelles and proteins [7, 19]. As observed in other tissues we hypothesized that autophagy could play an important role in regulating the maintenance of accumulated molecules with a subsequent effect on prostatic inflammation [7]. In our study we showed that autophagy is suppressed in the prostatic cells in the presence of a significant prostatic inflammatory infiltrates (IS > 7). Indeed in this group of patients we observed high expression of p62 and a low level of LC3B. Particularly the positive expression of p62 increased by ten times the risk of severe prostatic inflammation raising the question of the potential role of autophagy in inflammatory response in patients with LUTS/BPH. Thus inhibition of autophagy through different unknown mechanisms may activate the inflammatory response in the prostate by the increased expression of pro-inflammatory genes/cytokines and decreased expression of anti-inflammatory genes/cytokines as observed in the adipocytes or in pancreatic cells [7, 19]. Particularly, evidence from animal models indicates that autophagy is impaired in pancreatitis, and that one possible mechanism involved is the defective functions of lysosomes. As for prostatic inflammation in our study, pancreatitis decreases autophagy efficiency by an increased level of p62, a multifunctional protein that mediates autophagic clearance of ubiquitinated protein aggregate [11]. The p62 accumulation in autophagy-deficient cells also leads to NF-kb activation, clears apoptotic material which induces tissue inflammation or could increase levels of ROS which is required for inflammasone activation, a complex of cytosolic proteins, secerned by immune cells (macrophages and dendritic cells) in response to different “danger signals” which cleaves pro-IL-18 to the mature form and further increases its secretion from immune cells. Inflammasome, ROS and IL-8 activities have been recently associated to the activation of the prostatic associated lymphoid tissue and the development of prostatic inflammatory infiltrates [20–22] with a subsequent inflammatory tissue damages and continuative wound healing finally may induce the development of BPH nodules. Recent data also suggested a possible role of autophagy dysregulation in prostate cancer development and progression. Burdelski C et al. [23] in a immunohistochemistry tissue microarray study of 12,427 prostate cancers, demonstrated that strong cytoplasmic p62 staining was linked to high Gleason grade, advanced pathologic tumor stage and early PSA recurrence. Analysis of cytoplasmic accumulation of p62 was considered a strong predictor of an adverse prognostic behavior of prostate cancer. Considering the possible link between inflammation and prostate cancer development and progression [3] the role of autophagy could be twofold.

Although what we know is only the tip of a very large iceberg and the evidence on the molecular mechanism behind the relationship between autophagy and prostatic inflammation is quite primitive, elucidating these possible mechanisms could lead to the identification of new therapeutic targets acting to normalize the autophagy function.

We must acknowledge some limitations to our study: it is a single center study with a small number of patients. Furthermore, no specific serum or molecular markers of prostatic inflammation were used and the authophagy status was evaluating exclusively using the expression of autophagy markers p62 and LC3B. Although, inflammatory markers is an interesting topic as they could be used to better identify patients with prostatic inflammation, at this stage the question on what is the gold standard marker for prostatic inflammation continues to be debated and as a consequence no specific prostatic inflammatory markers are routinely available in our clinic.

Although detection of autophagosomes by electron microscopy is still regarded as the gold standard to detect autophagy in tissue, this method is time and cost consuming and restricted to the application on non formalin fixed and paraffin embedded tissue. Therefore we use two of the major autophagy proteins, which have been validated in different studies [6, 24].

Our results apply to this study (patients with BPH and LUTS resistant to medical therapy or with chronic urinary retention treated with TURP) and cannot be extended to all patients at risk for LUTS. Notwithstanding all these limitations, it is the first study investigating the relationship between the key structural authophagosomal proteins p62 and LC3B, involved in delivery of damaged proteins mitochondria to authophagosomes, and inflammatory infiltrates in patients with LUTS/BPH treated with TURP. The current pilot study suggests that autophagy is an important process in prostatic inflammatory infiltrates development and progression, and could be considered a new possible target for the management of prostatic disease. Immunohistochemical assessment of key autophagy proteins, such as p62 and LC3B, is feasible and their expression may identify a group of patients with severe prostatic inflammatory infiltrates. These findings should be confirmed by further larger series of patients with prostatic diseases and further studies should also evaluate deeper insight the possible link between autophagy defect and prostatic inflammation associated metabolic diseases such as obesity and metabolic syndrome as recently observed for pancreatic disorders [11].

## MATERIALS AND METHODS

### Patients

From April 2014 to September 2015, a consecutive series of patients treated in our center with monopolar TURP were prospectively included in this study. Indications for surgery were LUTS/BPH resistant to medical treatment and chronic urinary retention. Our Ethical Committee approved the study and all patients signed a dedicated informed consent. Exclusion criteria included history of bladder or prostate cancer, chronic prostatitis, bladder stones, urethral stenosis and neurological diseases. Age, co-morbidities, anthropometric parameters including body mass index (BMI) were recorded for all patients. At the baseline all men were evaluated with the IPSS, an uroflowmetry was also recorded. Additionally, prostate volume was evaluated by means of trans-rectal ultrasound. A series of TURP samples were included in this study and evaluated for the presence of prostatic inflammatory infiltrates and for the expression of the autophagy proteins p62 and LC3B.

### Prostatic inflammatory assessment

According to the standardized classification system of chronic prostatitis (CP-CPPS) proposed by Nickel et al. in 2001 [15], all TURP specimens were examined to define the grade (no inflammatory cells, mild inflammation with scattered inflammatory cells, moderate inflammation characterized by non-confluent lymphoid nodules, severe inflammation defined by large areas of confluent infiltrates) the anatomical location (glandular, periglandular and/or stromal), and the extent (focal <10%, multifocal 10-50%, diffuse >50%) of the prostatitis. The inflammatory score (IS score) was calculated as the sum of the three different histological inflammatory parameters (anatomical location, grade, and extent), each parameter ranges from 1 to 3. High IS score was defined as ≥ 7 [15].

### Immunohistochemical procedure and evaluation

Immunohistochemistry was performed as previously described [23]. Briefly, after deparaffinization, all sections were immunostained with a 1:200 dilution of the anti-LC3B (Cell Signaling, Lausen, Switzerland) and 1:200 of the anti-anti-p62/SQSTM1 antibodies (MBL, Nunningen, Switzerland). The primary antibody was omitted and replaced with preimmune serum in the negative control. Sections were reacted with biotinylated anti-rabbit antibody and streptavidin-biotin-peroxidase (Dako Laboratories, San Francisco, CA). Diaminobenzidine was used as a chromogene substrate. Finally, sections were washed in distilled water and weakly counterstained with Harry's modified hematoxylin. All sections were examined independently by two investigators (A.V., A.S.). Any positive reaction was scored as follow: 0 = No dots; 1 = detectable dots in 5-25% of cells; 2 = readily detectable dots in 25-75% of cells; 3 = dots in >75% of cells. High percentage of p62 or LC3B was defined as >25%, whereas low percentage of p62 or LC3B was defined as <25% of cells with dots.

**Figure 1:**
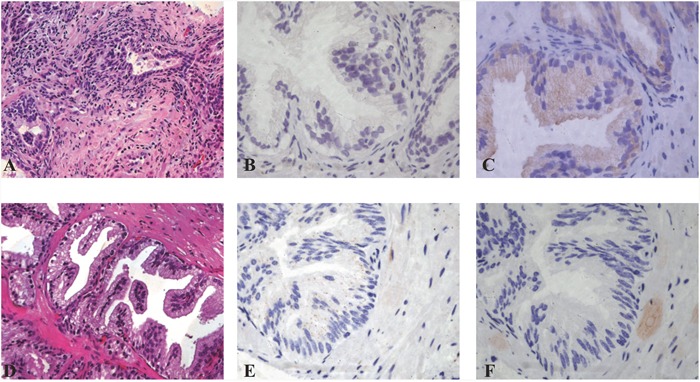
Staining of LC3B and P62 in prostate glands in different inflammatory conditions **A-C**. Inflammation score 8 prostatitis (**A**, 20x) showing LC3B negative staining (**B**, 40x) and P62 dot-like positive staining score 3 (**C**, 40x). **D-F**. Inflammation score 8 prostatitis (**D**, 20x) LC3B dot-like positive staining +2 score (**E**, 40x) and P62 negative staining (**F**, 40X) are shown.

The diffuse cytoplasmic reactivity of p62 antibodies and LC3B was assessed semi quantitatively. The intensity of the cytoplasmic pattern was evaluated as the proportion of cells with a strong, weak, or absent reactivity in all available optical fields of a tissue section at 20X magnification. The mean value was taken into consideration. Cases without any expression were considered as being negative. Thus, three distinct groups were created: (1) negative/weak expression, (2) strong expression in ≤50% of cells (limited over expression), and (3) strong expression in >50% of cells (extensive over expression). The percentage of cells with nuclear p62 expression was also assessed in all optical fields (magnification 20X). Cases with nuclear staining in >50% of cells were considered as positive; the remaining were recorded being negative (Figure 1).

### Statistical analysis

Statistical analysis was performed using the S-PSS 12.0 software. Evaluation of data distribution showed a non-normal distribution of the study data set. Differences between groups of patients in medians for quantitative variables and differences in distribution for categorical variables were tested with the Kruskal Wallis one-way analysis of variance and chi-square test, respectively. We conducted an uni-multivariate logistic regressions to assess the association between autophagy proteins expression and the overall risk of prostatic inflammation. The variables considered for entry into the model were age, p62 and LC3B (categorical variables). An alpha value of 5% was considered as threshold for significance. Data is presented as median [Inter quartile range (IQR), mean ± standard deviation (SD)].

## CONCLUSIONS

Here we present the first evidence of autophagy deregulation in prostatic inflammation. These results raise many questions about the “upstream” mechanisms mediating the autophagy dysfunction and the “downstream” links to prostatic inflammation that need to be addressed. Answers to these questions will provide new insight into molecular targets and therapeutic strategies for treatment of prostatic diseases.

## Abbreviations

LUTS: lower urinary tract symptoms
BPH: Benign Prostatic Hyperplasia
SQSTM1: sequestosome
TURP: TransUrethral Resection of Prostate
IS: inflammatory score
REDUCE: Reduction by Dutasteride of Prostate Cancer Events trial
CP-CPPS: classification system of chronic prostatitis
NIH: National Institutes of Health
IPSS: International Prostate Symptoms Score
MTOPS: Medical Therapies of Prostate Symptoms
BMI: body mass index
IQR: inter quartile range
SD: standard deviation
